# Chromosome segregation error during early cleavage in mouse pre-implantation embryo does not necessarily cause developmental failure after blastocyst stage

**DOI:** 10.1038/s41598-020-57817-x

**Published:** 2020-01-21

**Authors:** Daisuke Mashiko, Zenki Ikeda, Tatsuma Yao, Mikiko Tokoro, Noritaka Fukunaga, Yoshimasa Asada, Kazuo Yamagata

**Affiliations:** 1 Graduate School of Biology-Oriented Science and Technology, Kindai University, Wakayama, 649-6493 Japan; 2 Research and Development Center, Fuso Pharmaceutical Industries, Ltd., Osaka, 536-8523 Japan; 3 Asada Institute for Reproductive Medicine, Asada Ladies Clinic, Aichi, 450-0002 Japan

**Keywords:** Mitosis, Chromosome segregation

## Abstract

In the pre-implantation embryo, aneuploidy resulting from chromosome segregation error is considered responsible for pregnancy loss. However, only a few studies have examined the relationship between chromosome segregation errors during early cleavage and development. Here, we evaluated this relationship by live-cell imaging using the histone H2B-mCherry probe and subsequent single blastocyst transfer using mouse embryos obtained by *in vitro* fertilization. We showed that some embryos exhibiting early chromosomal segregation error and formation of micronuclei retained their developmental potential; however, the error affected the blastocyst/arrest ratio. Further, single-cell sequencing after live-cell imaging revealed that all embryos exhibiting micronuclei formation during 1^st^ mitosis showed aneuploidy at the 2-cell stage. These results suggest that early chromosome segregation error causing micronuclei formation affects ploidy and development to blastocyst but does not necessarily cause developmental failure after the blastocyst stage. Our result suggests the importance of the selection of embryos that have reached blastocysts.

## Introduction

In pre-implantation embryos, mosaic embryos, which are a mixture of both aneuploid and diploid cells, are frequently observed; 70% of human embryos and 25% of mouse embryos show chromosomal mosaicism^1–3^. This mosaicism is considered to be responsible for pregnancy loss^4–7^. A study using an aggregation chimeric mouse embryo of diploid blastomeres and drug-induced aneuploid blastomeres^8^ revealed that having a high percentage of aneuploid blastomeres affects embryo development at early post-implantation. An increased number of aneuploid blastomeres in embryos may result from chromosome segregation errors during early division. Based on this inference, Munné *et al*.^9^. estimated the time at which the error occurred based on the chromosome mosaic rate using the human embryo as an index. They suggested that in 50.7% of mosaic embryos, the abnormality originated during the first mitotic division, 25.7% in the second, and 23.6% in the third or later. However, few studies have evaluated the relationship between chromosome segregation errors in early cleavage and embryo development.

There are two types of chromosome segregation errors: chromosome misalignment during metaphase and lagging chromosomes during anaphase^10,11^. Furthermore, the severity of chromosomal segregation errors ranges from mild, where the chromosome deviates a few micrometers from the others, to severe with micronuclei formation. Using our less-invasive live-cell imaging system optimized for long-term imaging for the analysis of molecular dynamics of mammal pre-implantation embryos^12^, the type/severity of early chromosomal segregation errors in pre-implantation embryos can be assessed. We performed embryo transfer after fluorescence observation of chromosomal segregation using mouse embryos obtained by *in vitro* fertilization (IVF) (a technique where unfertilized eggs retrieved from females are fertilized *in vitro* with spermatozoa). Mixing the categorized embryos and then transferring them to recipient mice is challenging, and information other than the category will be lost, making detailed analysis difficult. Combining live-cell imaging and single embryo transfer could overcome this problem^13^, and we could directly link the relationship between the type/severity of the result of transplantation. Further, previous studies on the relationship between embryo ploidy and developmental potential used biopsy of blastocysts and subsequent chromosome analysis^14–16^; in this study, ploidy of blastomeres of 2-cell embryos was investigated by single-cell genome sequencing after live-cell imaging of 1^st^ mitosis to link the imaging data of chromosome segregation and ploidy of embryo. Through live-cell imaging, single embryo transfer, and genome sequencing at single-cell resolution, we demonstrated that early chromosomal segregation error resulting in aneuploidy in mouse pre-implantation embryos is a developmental risk to the blastocyst, but some blastocysts retain their developmental potential.

## Methods

### Animals

This study conformed to the Guide for the Care and Use of Laboratory Animals. All animal experiments were approved by the Animal Care and Use Committee at the Research Institute for Kindai University (permit number: KABT-31–016). ICR or B6D2F1 (BDF1) strain mice (12–16 weeks old) were obtained from Japan SLC, Inc. (Shizuoka, Japan). Room conditions were standardized, with the temperature maintained at 23 °C, relative humidity at 50%, and a 12-h/12-h light-dark cycle. Animals had free access to water and commercial food pellets. Mice used for experiments were sacrificed by cervical dislocation.

### *In vitro* fertilization (IVF)

IVF is a technique by which eggs retrieved from females are fertilized *in vitro* with spermatozoa. IVF was performed as described previously^12^. Females were superovulated by injecting 10 IU of pregnant mare serum gonadotropin (ASKA Pharmaceutical Co., Ltd., Tokyo, Japan; to bring the oocytes to maturity), followed by 10 IU of human chorionic gonadotropin (ASKA Pharmaceutical Co.; to release the oocytes) 48 h later. Ovulated oocytes were collected from the oviducts 14 h after human chorionic gonadotropin injection. Cumulus-enclosed oocytes were placed in 200-µL drops of TYH medium^17^ and covered with paraffin oil (Nacalai Tesque, Kyoto, Japan). Spermatozoa were collected by mechanically dissecting the cauda epididymites and were placed in 200-µL drops of TYH medium. After 2 h of incubation (in this duration, sperm gains the ability to fertilize), the sperm suspension in TYH was added to the TYH drop containing eggs at a concentration of 100 sperm/μL. After 2 h incubation at 37 °C under 6% CO_2_ in air (in this duration, spermatozoa fertilizes with oocytes), cumulus cells were dispersed by brief treatment with hyaluronidase (Type-IS, 150 U/mL, Sigma, St. Louis, MO, USA). Three hours after the dispersion of cumulus cells, the number of pronuclei was counted to check the fertilization (2 pronuclear embryos are the fertilized embryos).

### Live-cell imaging

The method used to prepare mRNA encoding histone H2B-mCherry was described previously^18^. Briefly, mRNA was prepared with the RiboMAX^TM^ Large-Scale RNA Production Systems- T7 (Promega, Madison, WI, USA). The 5′ end of mRNA was capped using a Ribo m7G Cap Analog kit (Promega) to prevent degradation. Synthesized RNA was purified by phenol-chloroform treatment and subsequent gel-filtration using a MicroSpin^TM^ S-200 HR column (GE Healthcare, Amersham, UK) to remove reaction intermediates and then stored at −80 °C until use. H2B-mCherry mRNA (5 ng/μL) was injected into the cytosol of the zygote at the pronuclear stage using a piezo manipulator in HEPES-buffered Chatot-Ziomek-Bavister medium^19^. The injected zygotes were transferred into 5-μL drops of KSOMaa medium^20,21^ containing 0.00025% polyvinyl alcohol and 100 µM EDTA on a film-bottom dish (Matsunami Glass Ind., Ltd., Osaka, Japan). Embryos were imaged three-dimensionally using a boxed type confocal laser microscope with an incubation chamber (CV1000, Yokogawa Electric Corp., Tokyo, Japan) set at 37 °C in 6% CO_2_, 5% O_2_, and 89% N_2_ with saturated humidity. We performed live-cell imaging four times (experimental replication number is 4).

### Embryo transfer

Embryo transfer was performed as described previously^13^. When we transferred analyzed single ICR blastocyst, 6 BDF2 strain blastocysts produced by routine IVF were co-transferred as carrier blastocysts to maintain pregnancy in the recipient females. These carrier blastocysts and analyzed single ICR blastocysts were transferred into unilateral 2 days post-coitum uteri (Mice have a bicornuate uterus, and bilateral transfer is associated with the risk of loss of correspondence by moving the embryo to the opposite side). To avoid natural births, recipient females were sacrificed at 18.5 days post-coitum to evaluate the full-term developmental ability of each analyzed embryo. Mice derived from analyzed embryos were distinguished by their eye colors; briefly, analyzed mice had red eyes, and mice derived from carrier blastocysts had black eyes.

### Single-cell/whole-blastocyst genome sequencing

After observing the 1^st^ mitosis by live-cell imaging, 2-cells were recovered from the zona pellucida using a piezo manipulator^22^ and then incubated for 10 min in calcium- and magnesium-free PBS. Each blastomere and polar body were separated by gently pipetting, and each blastomere was collected. Blastocysts were collected after live-cell imaging. An Ion ReproSeq PGS kit was used for extraction, amplification, and barcoding of genomic DNA (A34899, Thermo Fisher Scientific, Waltham, MA, USA). The barcoded samples were pooled and evaluated on the Ion Chef System and Ion S5 Next-generation sequencing system (Thermo Fisher Scientific). The resulting reads were mapped to the mouse reference genome (mm9) using Bowtie software (http://bowtie-bio.sourceforge.net). We then calculated the moving average of mapping read counts within a 10-Mbp window. Moving averages were compared in all groups for each chromosome to identify the chromosomal defect. Circos plots summarizing ploidy were created in R software as described by Bolton *et al*.^8^: “Each circos plot represents the genomic constitution of an embryo in all its cells, with blastomeres presented as rings and the chromosomes as segments.”

### Statistical analyses

Chi-square/prop test for ratio analysis and Ryan’s method for multiple testing to determine the blastocyst/arrest or born/abort ratio were performed using R software (https://www.r-project.org/). A P-value > 0.05 was considered not significant (n.s.), whereas P-values < 0.05 (*), < 0.01 (**), and < 0.001 (***) were considered significant.

## Results

### Live-cell imaging of chromosome segregation and characterization of the severity of chromosome segregation error

**Figure 1:**
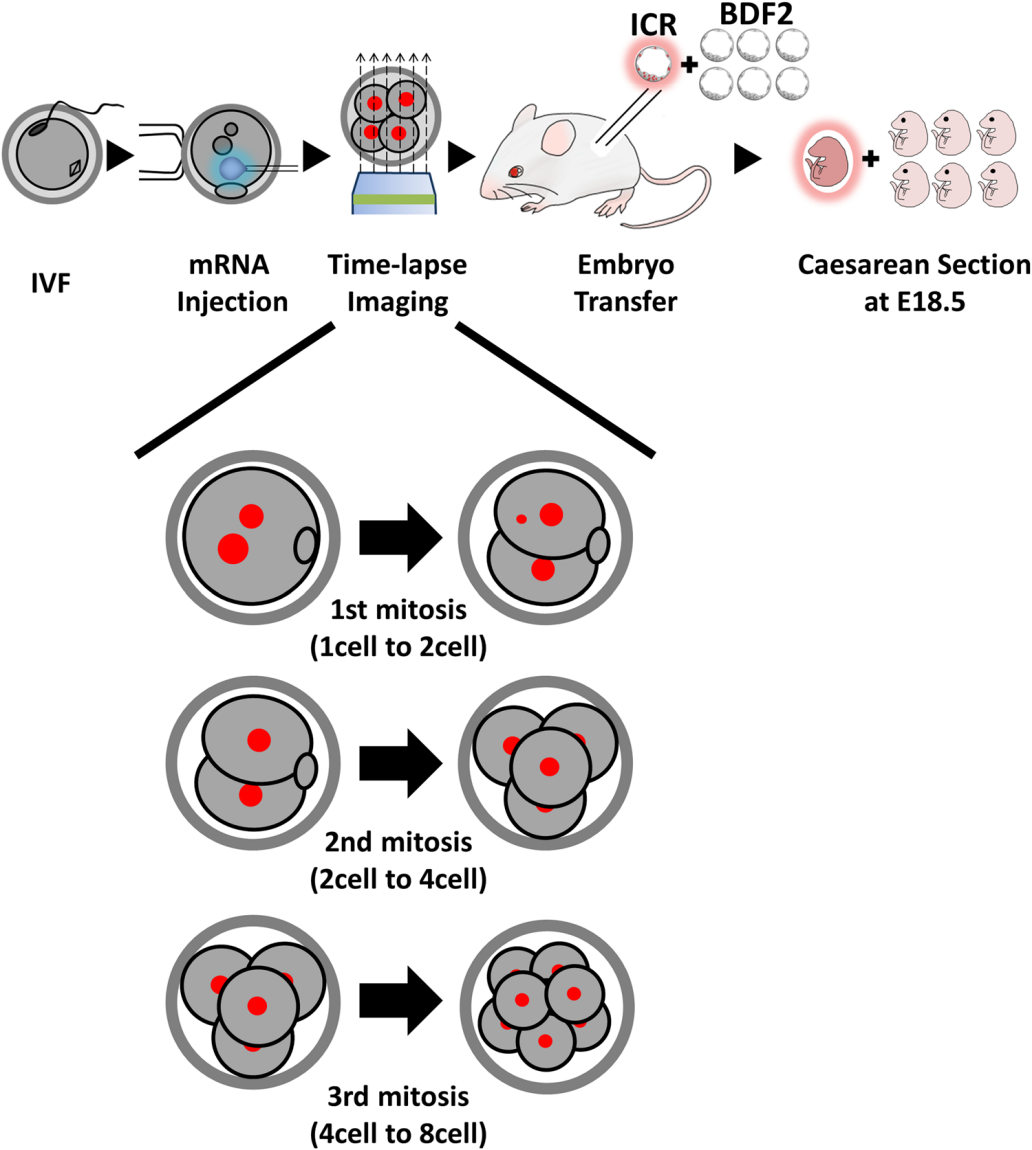
Schematic diagrams of live-cell imaging and subsequent embryo transfer. We performed IVF to obtain pronuclei (PN) stage embryos. After injecting mRNA encoding histone H2B-mCherry into the cytosol of 2PN, time-lapse imaging was performed 3-dimensionally. We observed chromosome segregation during the 1^st^, 2^nd^, and 3^rd^ mitosis. Single embryo transfer of observed blastocysts was performed on day 4. Six blastocysts with different coat colors were implanted as controls to determine whether the case of no pups was due to embryo quality or pseudopregnant mouse implantation ability/technique of embryo transfer. Sixteen days after embryonic transfer, pups were obtained by cesarean section.

To observe chromosome segregation, mRNA encoding mCherry fused with histone H2B (H2B-mCherry) was injected into pronuclear stage ICR embryos generated by IVF. We observed the embryonic development up to the blastocyst and retrospectively characterized abnormal chromosome segregation (ACS) timing and severity during early mitosis. Among the observed embryos (n = 231), 194 embryos reached the blastocyst stage (Thirty embryos were morula, and 7 embryos were cleavage stage at day 4). Seventy-five blastocysts were randomly picked, and one observed blastocyst was transferred into one pseudopregnant mouse. As a control for the technique and to determine the implantation ability of pseudopregnant mice, six embryos from strains with different coat colors (BDF2 strain) were simultaneously transferred. These embryos were identified by examining the eye colors of the offspring (Fig. 1).

**Figure 2:**
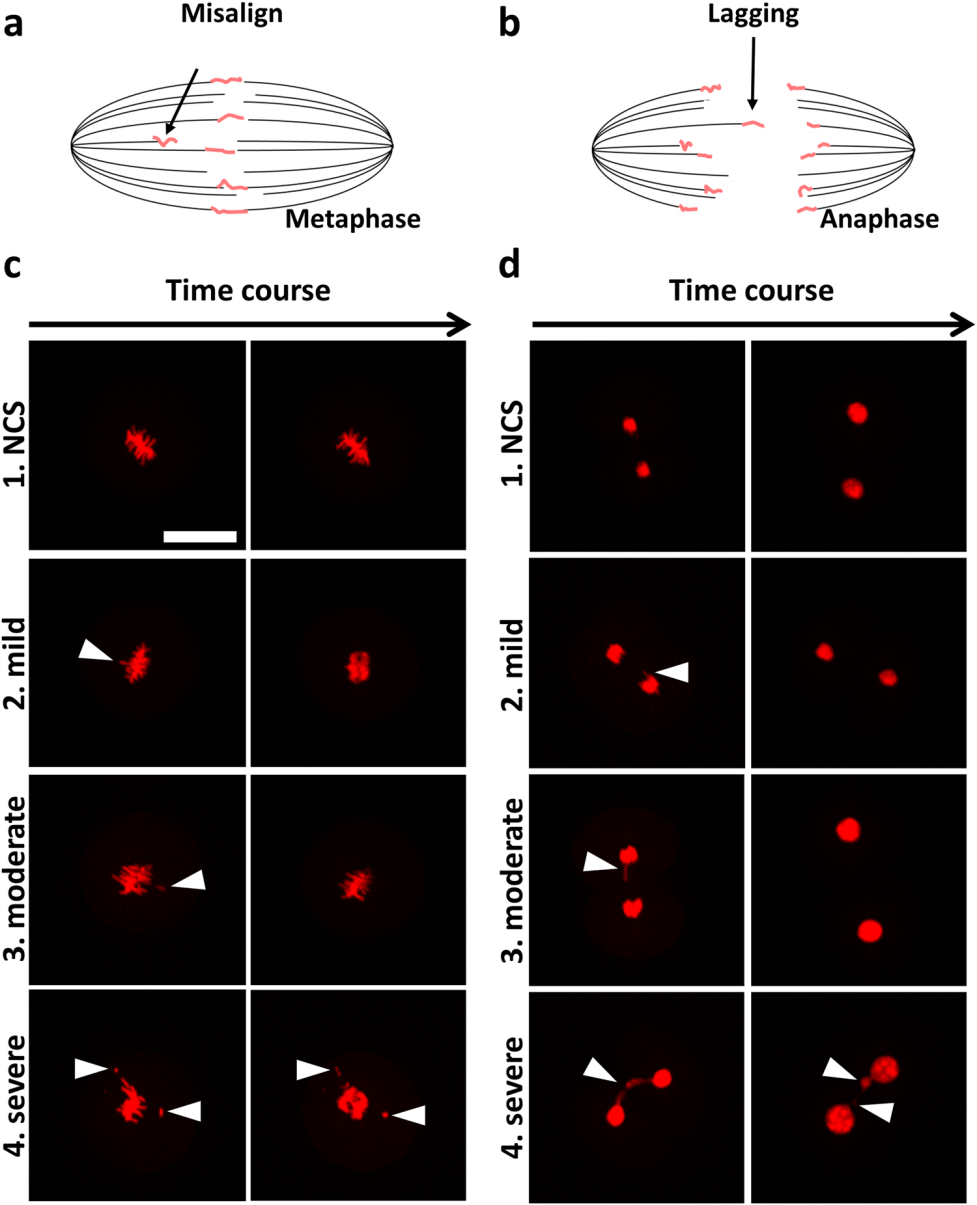
Categorization of severity and type of chromosome segregation error. The categorization of chromosome segregation error based on imaging. There are two types of chromosome segregation errors: misalignment at metaphase **(a)** and lagging chromosome at anaphase **(b)**. Severity of chromosome segregation errors were categorized as follows, 1: normal chromosome segregation (NCS), 2: one or more chromosomes deviate a few micrometers from others and return, 3: one or more chromosomes deviate completely from others and return, 4: one or more chromosomes deviate completely from others and form the micronucleus. The typical snapshots of each severity of misaligned and lagging chromosomes are shown in **(c)** and **(d)**. Bar = 50 μm.

When chromosome segregation was monitored, misaligned (Fig. 2a) and lagging (Fig. 2b) chromosomes were observed. Since these segregation errors ranged from mild errors in which micronuclei were not formed to severe errors in which micronuclei were formed, we classified the severity of errors in chromosome segregation into the following four stages (Fig. 2c,d): 1. Normal chromosome segregation (NCS), 2. mild error (chromosome deviated a few micrometers from the others), 3. moderate error (chromosome lagged/misaligned from the others completely at least 20 min), and 4. severe error which formed micronuclei; we referred to this state as ACS in our previous study^13,23^).

### Micronuclei during early cleavage affect the development to the blastocyst

**Figure 3:**
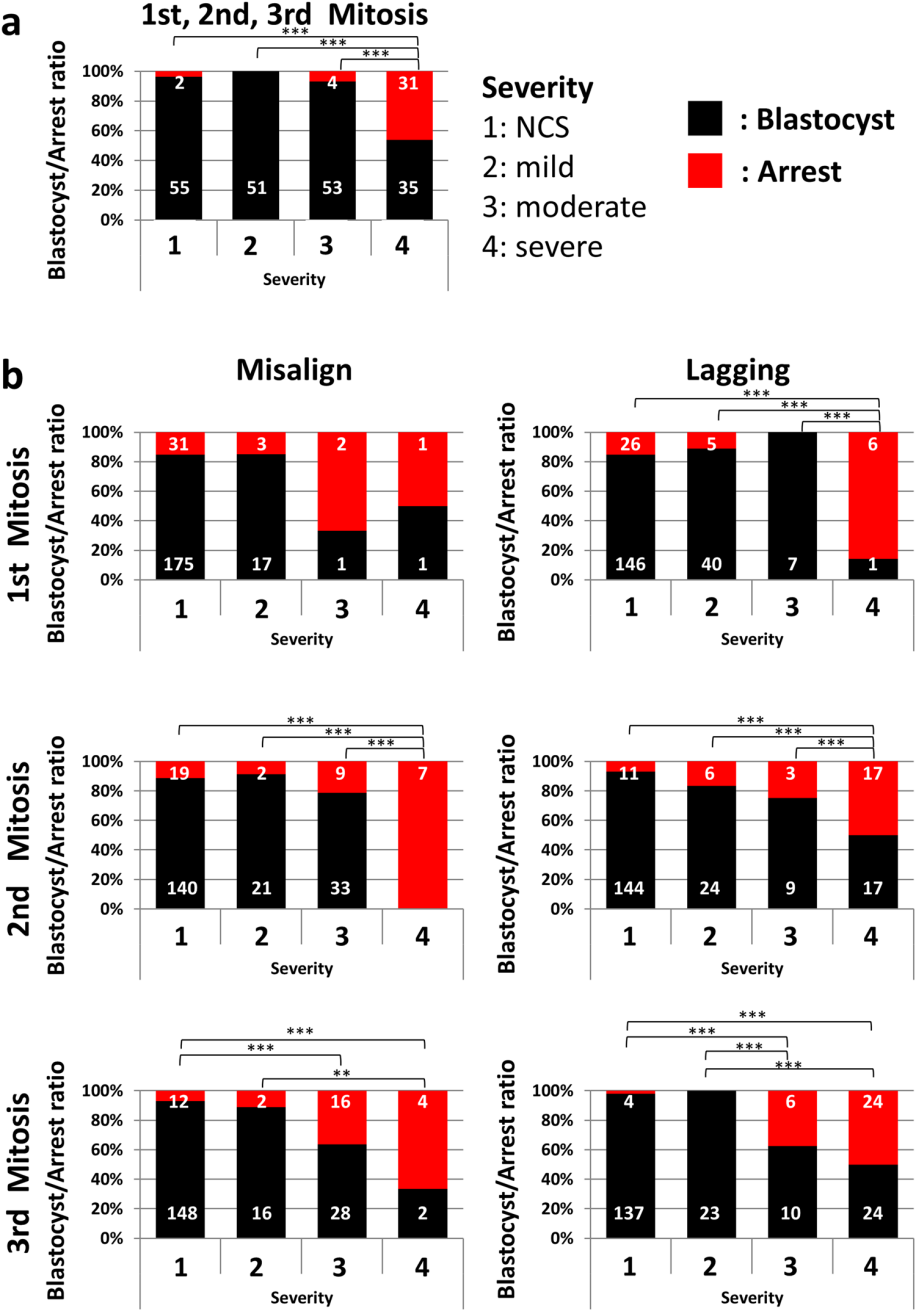
Chromosome segregation error forming micronucleus affects blastocyst/arrest ratio. (**a**) Cumulative bar plot showing the relationship between blastocyst/arrest ratio and most severe chromosome segregation error by 3^rd^ mitosis. The numbers in the bar plot show the numbers of embryos. **(b)** Cumulative bar plot showing the relationship between type/severity of chromosome segregation error during 1^st^, 2^nd^, and 3^rd^ mitosis and blastocyst/arrest ratio. As a result of power analysis, power = 0.8 for 3b.

We investigated the relationship between the most severe chromosome segregation error, which occurred by the 3^rd^ mitosis (maximum severity of errors in 1^st^, 2^nd^, and 3^rd^ mitosis) and the blastocyst rate (Fig. 3a). The blastocyst rate in each severity group of chromosome segregation errors was as follows: severity 1 (NCS): 55/57 (96.5%), severity 2 (mild error): 51/51 (100%), severity 3 (moderate error): 53/57 (93.0%), severity 4 (ACS): 35/66 (53.0%). The embryos categorized as severity 4 showed a significantly lower blastocyst rate than that in the other groups (Ryan’s method, P < 0.001). To reveal when and what types of errors affect development, we observed in detail whether the chromosome segregation error occurred during the 1^st^, 2^nd^, or 3^rd^ mitosis and whether the error type was misalign/lagging (Fig. 3b). As a result, regardless of the timing and type of error, embryos showing severity 4 error showed a low blastocyst/arrest ratio (Ryan’s method, P < 0.01; the number of embryos showing misalign at the 1^st^ mitosis was small and could not be analyzed statistically). During the 3^rd^ mitosis, in both misalignment and lagging cases, embryos causing severity 3 error showed a significantly lower blastocyst rate than those with severity 1 or 2 (Ryan’s method, P < 0.05, Fig. 3b). We investigated whether this result suggests a spurious correlation, which is caused by severity 4 error. Embryos showing micronuclei in 1^st^/2^nd^ mitosis; those showing severity 3 defects in the 3^rd^ mitosis had a ratio of 24/54 (44.4%, Supplemental Table 1), which was significantly higher than the percentage of severity 4 in early cleavage (66/231, 29.0%, Fig. 3a, Supplemental Table 1; Chi-square test, P < 0.05). A possible cause of the significant difference in arrest rate between embryos showing severity 3 defects in the 3^rd^ mitosis and those in earlier divisions is the accumulation of malfunction of the chromosome segregation mechanism by chromosome segregation error at 1^st^/2^nd^ mitosis. The malfunction of the chromosome segregation mechanism which causes multiple errors may indicate a problem in cell division per se, which may affect further development. In order to investigate the adverse effect of multiple errors on development, we analyzed the blastocyst/arrest ratio of embryos with unique errors and those with multiple errors. Regardless of whether it was unique or multiple, embryos that carried a severity 4 error had a low blastocyst rate (Ryan method and prop-test, P < 0.01, P < 0.001; Supplemental Fig. 1).

### Chromosome segregation error during 2^nd^, 3^rd^ mitosis does not cause pregnancy loss

**Figure 4:**
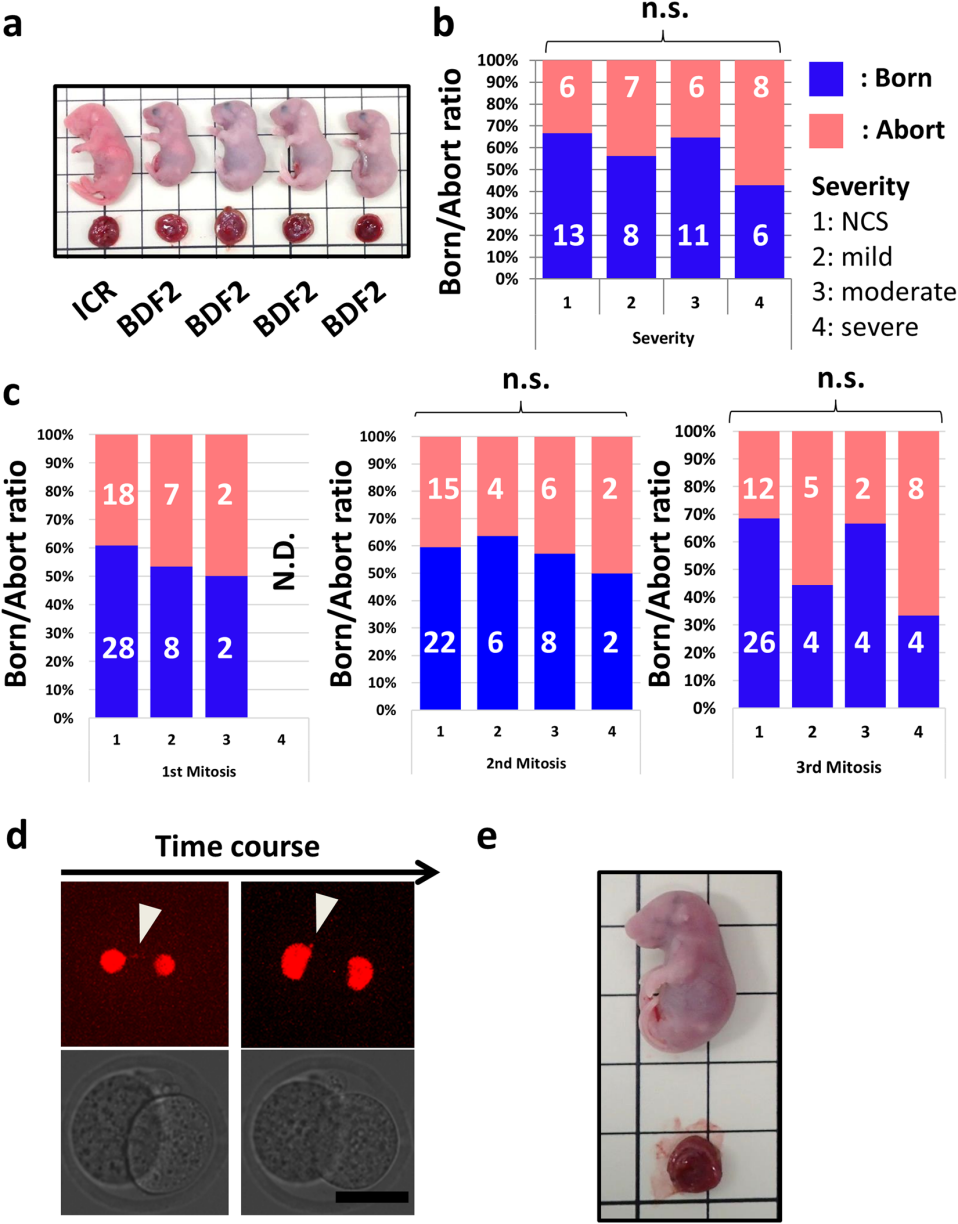
Chromosome segregation error does not affect the result of blastocyst transfer. (**a**) Photograph of the result of cesarean section. The eye color was used to determine if an observed embryo was obtained. **(b)** Cumulative bar plot showing the relationship between born/abort ratio and most severe chromosome segregation error by 3^rd^ mitosis. **(c)** Cumulative bar plot showing the relationship between severity of chromosome segregation error during 1^st^, 2^nd^, and 3^rd^ mitosis and born/abort ratio. **(d)** Snapshot of “Born” case embryo showing micronuclei (arrowhead) at 1^st^ mitosis. Bar = 50 µm. **(e)** Photograph of a pup from embryo showing micronuclei at 1^st^ mitosis.

After transferring the single observed ICR blastocyst to pseudopregnant mice, we performed a cesarean section to obtain offspring (Figs. 1 and 4a). When offspring derived from observed ICR embryos were obtained, the cases were treated as “born” (n = 38; Fig. 4a shows the “born” case). When offspring derived from BDF2 embryos were obtained, and those derived from observed ICR embryos were not obtained, the cases were treated as “aborted” (n = 27). Cases in which offspring were not obtained from either the embryos, which were the controls of transplantation (BDF2) or observed embryo (ICR), were considered to have problems with the technique or implantation ability of pseudopregnant mice and were excluded from analysis (n = 10). When we examined the relationship between the most severe chromosomal segregation error by the 3^rd^ mitosis and born ratio, there was no significant difference between groups (Fig. 4b; chi-square test, P = 0.61). For detailed analysis, we classified the results shown in Fig. 4b into 3 categories (i.e. 1^st^, 2^nd^, 3^rd^ mitosis; Fig. 4c). Since blastocysts showing severity 4 error during the 1^st^ mitosis were extremely rare (1/231: Fig. 3b), the blastocyst for transfer was not chosen by random draw. There was no significant difference between any groups in the 2^nd^ and 3^rd^ mitosis (chi-square test, P = 0.99, 0.13, Fig. 4c).

To confirm the impact of severity 4 at the 1^st^ mitosis on full-term development, we performed an additional experiment using 245 embryos. Nine of the 245 embryos (3.7%) showed severity 4 error at 1^st^ mitosis, and only 2 embryos (0.8%) showing severity 4 at the 1^st^ mitosis reached the blastocyst stage. These two blastocysts were transplanted into pseudopregnant mice. Finally, we obtained a pup from an embryo that had formed a micronucleus during the 1^st^ mitosis (Fig. 4d, e, Supplemental Movie 1). As far as the movie is concerned, we did not observe that micronuclei of born case embryo get into the nucleus but contact with nucleus (Supplemental Movie 2).

### Single-cell genome sequencing after live-cell imaging revealed a relationship between ploidy and chromosome segregation

**Figure 5:**
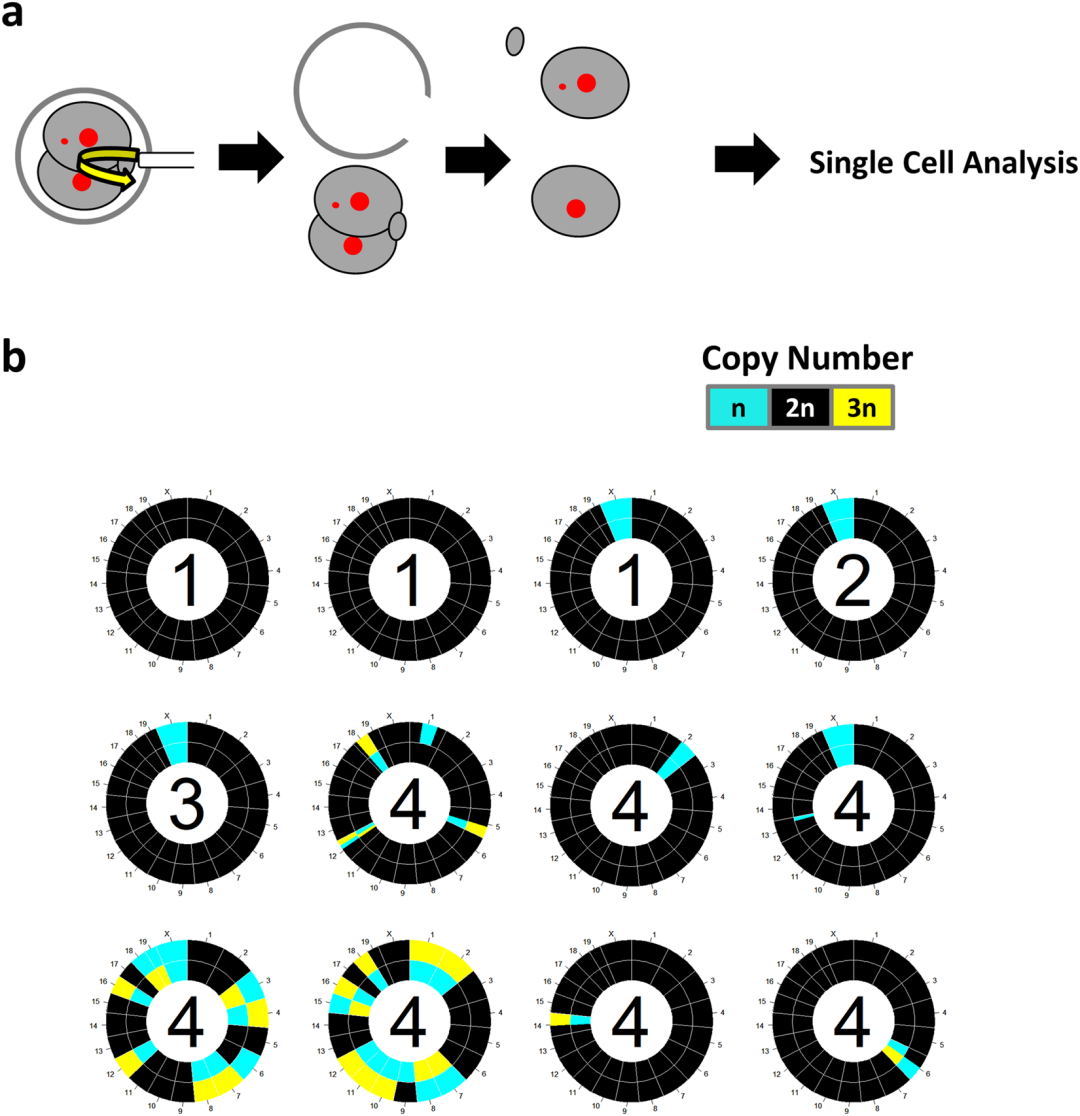
Relationship between chromosome segregation and ploidy (**a**) Schematic diagrams of blastomere collection. **(b)** Each circos plot shows the ploidy of a 2-cell stage embryo in all of its cells, with blastomeres presented as rings and chromosomes as segments. The copy number of X depends on the gender of the mouse. Inside of the circos plot, the severity of chromosome segregation error is shown.

Microscopic observation revealed the embryo showing micronuclei have a lower blastocyst rate than the embryos not showing micronuclei formation. Furthermore, we revealed that pups could be obtained even from embryos that formed micronuclei during 1^st^ mitosis, so we focused on the relationship between 1^st^ mitosis error and ploidy. Subsequently, we investigated how does the microscopic observation of chromosome segregation reflect ploidy. In addition, we tested whether the contact between the nucleus and micronuclei meant re-integrate. To clarify the relationship between chromosome segregation and embryo ploidy, after observing chromosomal segregation during the 1^st^ mitosis (Supplemental Movie 3), we recovered 2-cell embryos from the zona pellucida and examined the ploidy of each blastomere (Fig. 5a) by single-cell genome sequencing. Embryos showing chromosome segregation without micronuclei formation were euploid (Fig. 5b and Supplemental Table 2). In contrast, all embryos showing severe chromosome segregation errors (forming micronuclei) were aneuploid. Three of the seven embryos showing severity 4 error showed aneuploidy with multiple loci (42.9%), while 4 of 7 showed single locus aneuploidy (57.1%). Further, 6 of 7 (85.7%) embryos showed aneuploidy in both the blastomere; in 1 of 7 (14.3%) embryos, a blastomere on one side was euploid. In the analyzed embryos, there was an embryo with a micronucleus that was in contact with the nucleus (Supplemental Movie 4: this embryo shows the chr2 deletion in both blastomeres, Fig. 5b). Since this pattern of embryo also shows aneuploidy, contact of micronuclei with the nucleus does not necessarily mean re-integration. Here, we clarified that chromosome segregation error showing micronuclei causes aneuploidy.

### Whole-genome sequencing of blastocyst after live-cell imaging

We have shown that chromosomal segregation errors that form micronuclei during 1^st^ mitosis cause aneuploidy at the 2-cell stage. If an embryo that formed a micronucleus during 1^st^ mitosis grows up to the blastocyst stage, it should show 50–100% aneuploidy. Are embryos in category 4 that make it to the blastocyst stage those with a predicted value of aneuploidy? We observed 120 embryos and found two embryos in category 4 that made it to the blastocyst stage. We performed whole-genome sequencing of these embryos and predicted the mosaic rate by read-count. One embryo showed a 50% mosaic rate and the other embryo showed a 15% mosaic rate (Supplemental Fig. 2). This result suggests a decrease in abnormal cells in an embryo in the process of developing to the blastocyst.

## Discussion

### Relationship between micronuclei formation and blastocyst/arrest ratio

Micronuclei formation during early cleavage significantly affected the blastocyst/arrest ratio (Fig. 3a, b). Furthermore, embryos showed aneuploidy when the micronuclei were formed (Fig. 5b). These results suggest that aneuploidy affects the blastocyst/arrest ratio. Our results are consistent with a large-scale experiment in which biopsy was performed on human embryos prior to compaction, showing that the blastocyst/arrest ratio of mosaic embryos was significantly lower than that of euploid embryos^6^. The high failure rate in reaching the blastocyst stage in human embryo development (50–70% of embryos fail to reach the blastocyst stage *in vitro*; 24) may be related to severe chromosome segregation error during early cleavage.

### Chromosome segregation errors during early division do not necessarily cause pregnancy loss

In contrast to the negative effect of micronuclei formation on the blastocyst/arrest ratio, when embryos that had developed up to blastocysts were transplanted, no relationship was found between chromosomal segregation errors by the 3^rd^ mitosis and the born ratio (Fig. 4b, c). We are cautious about concluding the relationship between birth rate and severity of error because of the small number of transferred embryos. Here, we conclude that segregation errors are not necessarily detrimental to development. Since a chromosome segregation error, even in the 1st mitosis did not necessarily cause pregnancy loss, we hypothesized that some 2-cell embryos showing chromosome segregation error during the 1^st^ mitosis had at least one euploid blastomere. In a previous study in which mouse embryos were artificially induced to aneuploidy and chimeric embryos were prepared, 100% aneuploid embryos showed early post-implantation failure, and 50% of aneuploid embryos showed no problems and pups were obtained^8^. Additionally, in human embryos, a healthy baby was obtained from 50% of mosaic aneuploid blastocysts^15^. If one blastomere of the two-cell stage embryos was euploid, offspring were obtained if it caused chromosomal segregation error during the 1^st^ mitosis. In fact, 50% aneuploid mosaicism was detected in embryos that formed micronuclei during the 1^st^ mitosis (Fig. 5b). In addition, two embryos in category 4 at 1^st^ mitosis that made it to the blastocyst stage showed 50% and 15% mosaic rate (Supplemental Fig. 2). The embryo showing a 15% mosaic rate suggests the elimination of abnormal cells in the process of developing to the blastocyst.

Several studies using human and mouse embryos have suggested that the mosaic aneuploid ratio affects embryo development post-implantation^8,24^. In the present study, the adverse effects of early chromosome segregation errors that will change the ploidy on post-implantation development were not observed. There may be a threshold for the mosaic aneuploid ratio that adversely affects embryo development post-implantation, which was not exceeded by early chromosomal segregation error alone. The full-term developed embryo forming micronuclei at the 1^st^ mitosis did not cause additional errors in the 2^nd^ and 3^rd^ mitosis (Supplemental Movie 1). Since severity 4 error in 1^st^ mitosis rarely occurs in mice IVF embryos, it may be useful to consider the relationship between the mosaic aneuploid ratio and embryo development post-implantation using another method. In addition, in the future, it is essential to observe the fate of each blastomere (i.e. inner cell mass, trophectoderm, or dead). In the present study, we found that chromosome segregation errors that occur during early division do not necessarily cause pregnancy loss.

### Comparison of previous studies investigating intracytoplasmic sperm injection (ICSI)/or somatic cell nuclear transfer (SCNT) embryos showing abnormal chromosome segregation (ACS) and present study investigating IVF embryos showing ACS

Previous studies showed that the proportion of ACS is higher in ICSI embryos or SCNT embryos than in IVF embryos. In SCNT embryos, pups were not obtained from embryos showing ACS by the 3^rd^ mitosis^13^. After transplantation of ICSI embryos, only 2 pups were obtained from 45 embryos showing ACS in the 1^st^ mitosis (4.4%)^23^. Two previous studies suggested that ACS affects full-term development. However, these studies have not identified which stage of the embryo is adversely affected by ACS. These studies used 2-cell embryos or day 3 mouse embryos for transplantation to evaluate whether they affect the full-term development of ICSI/SCNT embryos. During day 3, most embryos were morulae, and some embryos reached the blastocyst stage. Therefore, previous studies have performed transplantation before the embryo reaches the blastocyst and have concluded that morphology of morulae and rate of development to morulae are not significant predictive markers for full-term development. In the present study, we used mouse day 4 embryos, which were obtained by IVF to evaluate whether the embryos reached the blastocyst stage; we transferred the blastocysts into the uteri to evaluate their developmental potential post-implantation. Further, among the embryos that stopped developing at the morula stage (n = 30), most embryos (n = 25) showed severity 4 error (Supplemental Table 1). Here, we hypothesize that almost no pups were obtained from ICSI/SCNT embryos because ACS affected the blastocyst/arrest ratio even in the previous two studies. To improve the results of transplantation of ICSI/SCNT embryos, it may be effective to select blastocyst transfer than 2-cell transfer/morula transfer.

### Proposal for new ploidy test technology

The relationship between early chromosome segregation error and ploidy (Fig. 5b) suggests that it is possible to test for karyotypes without a biopsy. Biopsy is a widely used method for testing embryos but may affect pregnancy rates^14^. In addition, the current biopsy technique collecting multiple blastomeres of the blastocyst is not suitable to detect an error as it provides the average of copy number, which could be 2n even when there is an error as detected in the present study (i.e. one blastomere is n, and the other is 3n: Fig. 5b). Our results and the success of pregnancy after time-lapse imaging in the mouse/bovine embryo^12,25^ suggest that karyotyping without biopsy can be applied to livestock embryos. In contrast, our technology involves injecting mRNA encoding fluorescent protein and laser irradiation for excitation, making it difficult to directly apply to human embryos. The correlation between blastomere behavior and human ploidy^26^ will be useful for predicting aneuploidy (e.g. this correlation would obviously exclude abnormal embryos), but direct observation of micronuclei formation is more accurate in the karyotype test. Methods for observing micronucleus formation by bright-field observation will enable non-invasive karyotype examination of human embryos.

### Strategies for selecting embryos for transplantation

In the present study, we observed a relationship between chromosome segregation and the resulting aneuploidy, and that chromosome segregation error during early cleavage affects embryonic development. The relationship between early chromosome segregation error and the blastocyst/arrest ratio (Fig. 3a, b) suggests that cleavage-stage embryos that will reach the blastocyst stage can be selected. In this study, we showed that the blastocyst/arrest ratio of embryos forming micronuclei during early cleavage was lower than that of other embryos. Therefore, by observing micronuclei, the risk of decreasing the blastocyst rate can be avoided. The presence of micronuclei can be used as a selection indicator for transfer during early cleavage stage embryos into the mother. In contrast, the birth rate of embryos that developed to the blastocyst stage even with micronuclei formation during the 2^nd^ or 3^rd^ mitosis did not differ from that of the other embryos (Fig. 4b, c). Further, we even obtained offspring from a blastocyst showing chromosome segregation error in the 1^st^ mitosis (Fig. 4e). Although the risk of chromosomal disease remains and it may be not enough to filter out potentially defective embryos, it may be possible to decrease the risk of pregnancy loss due to embryonic arrest during early mitosis by transplanting embryos that have developed up to blastocysts. Our results support the blastocyst transfer recommended in the clinic^27^ in terms of avoiding aneuploidy.

## Supplementary information

Supplemental information.

The ‘born’ embryo with 1st mitosis micronuclei.

Three-dimensional reconstruction of the ‘born’ embryo with a 1st mitosis micronuclei.

The 1st mitosis of the embryos that were investigated by single-cell sequencing.

Three-dimensional reconstruction of a severity 4 embryo that micronuclei are in contact with the nucleus.

Supplemental_Table1 The severity of observed embryos.

Supplemental_Table2 Analyzed data of single-cell sequencing.

## Data Availability

All data generated or analyzed during this study are included in this published article and its supplementary information files.
