# Supplementary material for: Chromosome segregation error during early cleavage in mouse pre-implantation embryo does not necessarily cause developmental failure after blastocyst stage
Source: Sci Rep. 2020 Jan 21;10:854. doi: 10.1038/s41598-020-57817-x (PMC6972754; doi:10.1038/s41598-020-57817-x)
Supplement: Supplementary file 1 — Supplemental information. [file 41598_2020_57817_MOESM1_ESM.pdf]

Supplementary information

Chromosome segregation error during early cleavage in mouse pre-implantation embryo does not necessarily cause developmental failure after blastocyst stage

Daisuke Mashiko, Zenki Ikeda, Tatsuma Yao, Mikiko Tokoro, Noritaka Fukunaga, Yoshimasa Asada, Kazuo Yamagata

Supplemental Figure. 1

The formation of micronuclei, not multiple errors, is related to developmental arrest.

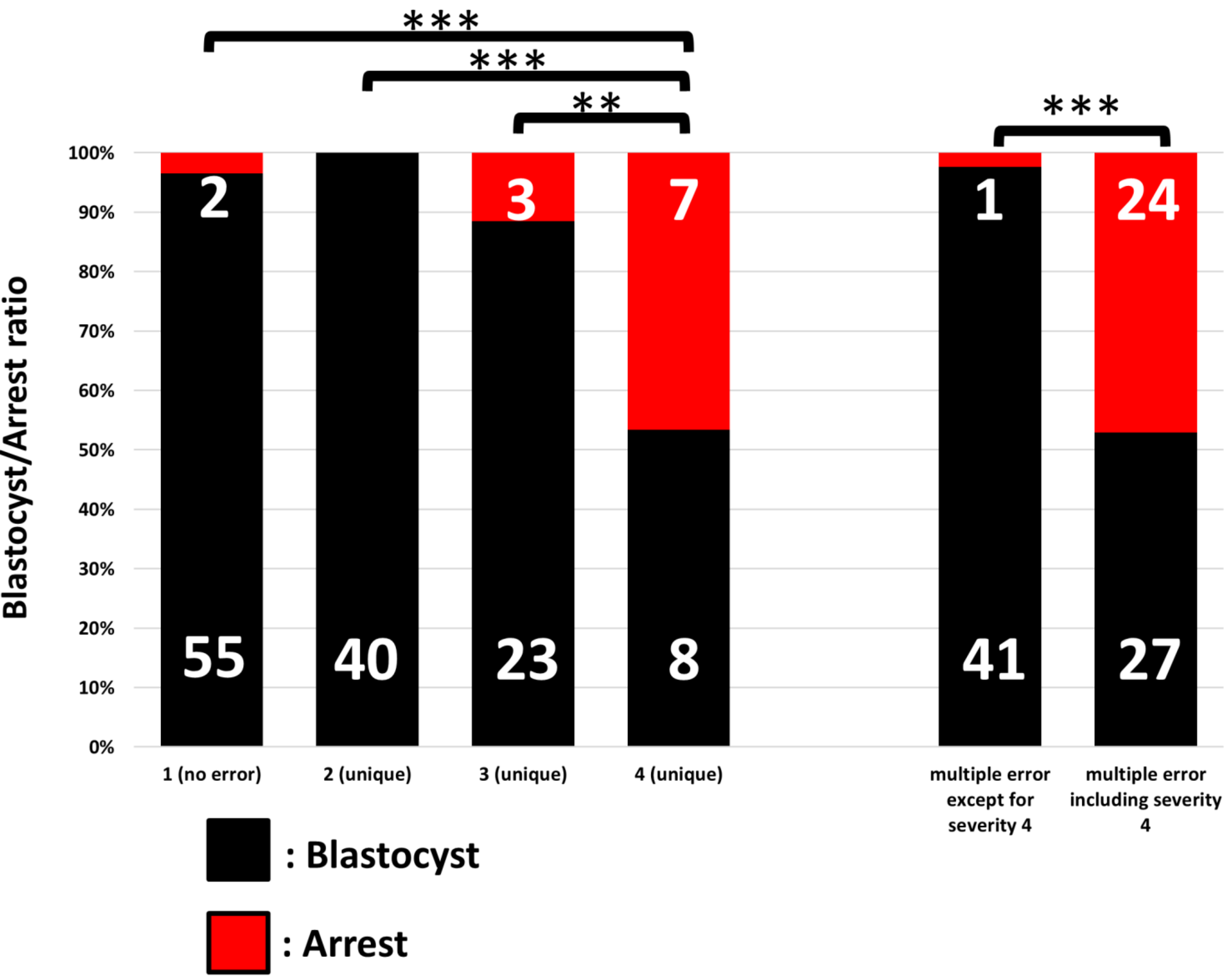

Supplemental Figure. 1

The formation of micronuclei, not multiple errors, is related to developmental arrest.

Each bar shows the blastocyst/arrest ratio. From left to right, each bar shows "no error during early mitosis", "only one error which is severity 2 during early mitosis", "only one error which is severity 3 during early mitosis", "only one error which is severity 4 during early mitosis", "segregation error in more than one early division except for severity 4 error", "segregation error in more than one early division including severity 4 error".

Supplemental Figure. 2

Whole-genome sequencing of blastocyst after live-cell imaging

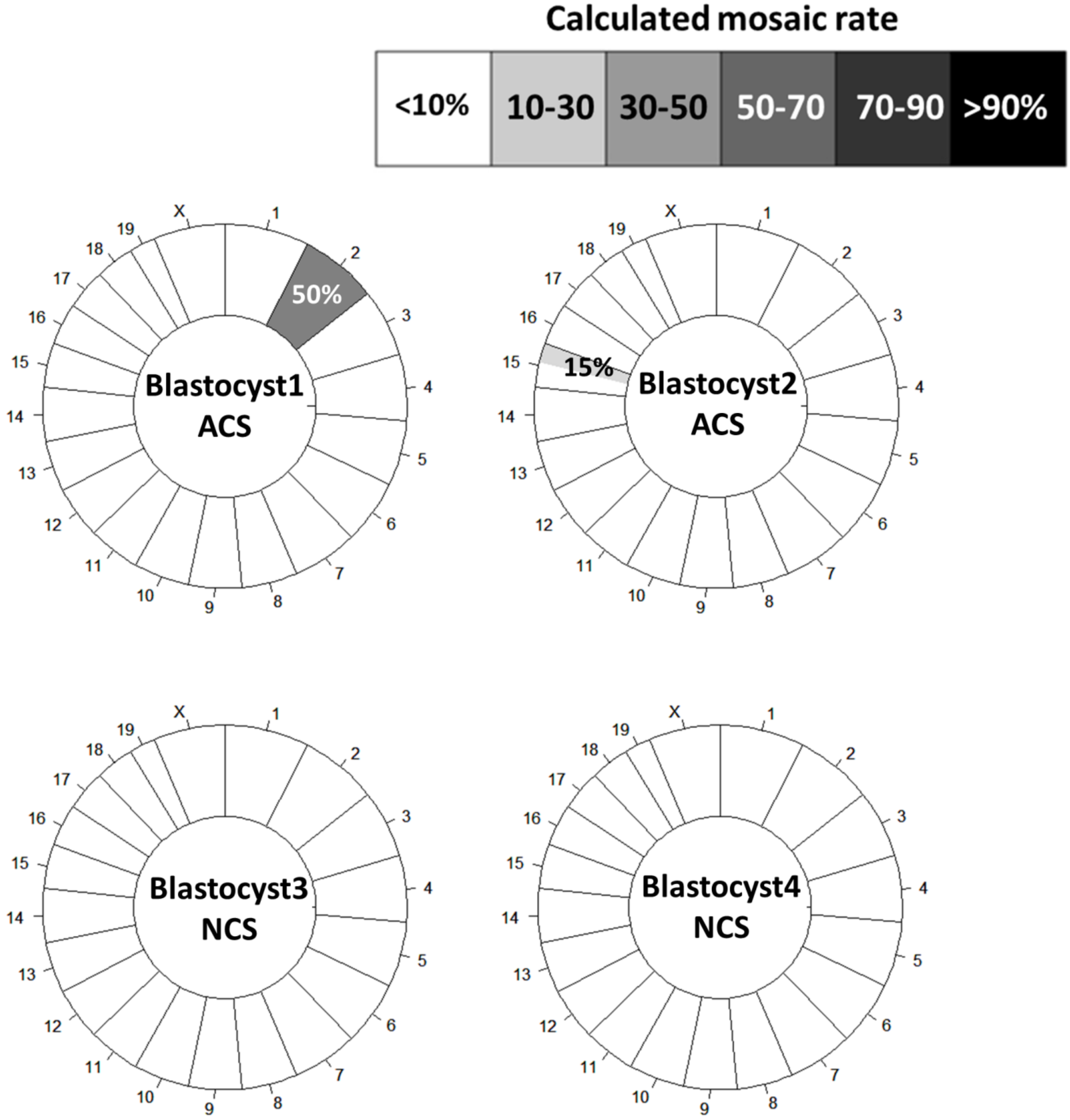

Supplemental Figure. 2

Whole-genome sequencing of blastocyst after live-cell imaging

Upper charts show the calculated mosaic rate of blastocysts that showed micronuclei formation at 1st mitosis.
Bottom charts show the calculated mosaic rate of blastocysts, which did not show chromosome segregation error by 3rd mitosis.
The mosaic rates were calculated based on the read count.
